# Case Report: Identification of a novel *STAT3* mutation in EBV-positive inflammatory follicular dendritic cell sarcoma

**DOI:** 10.3389/fonc.2023.1266897

**Published:** 2023-10-26

**Authors:** Megan C. Ramsey, Peter J. B. Sabatini, Geoffrey Watson, Tanya Chawla, Michael Ko, Ali Sakhdari

**Affiliations:** ^1^ Hematopathology Department, Toronto General Hospital, Toronto, ON, Canada; ^2^ Department of Laboratory Medicine and Pathobiology, University of Toronto, Toronto, ON, Canada; ^3^ Division of Clinical Laboratory Genetics, Laboratory Medicine Program, University Health Network, Toronto, ON, Canada; ^4^ Advanced Molecular Diagnostic Laboratory, Princess Margaret Cancer Centre, Toronto, ON, Canada; ^5^ Medical Oncology, Mount Sinai Hospital, Toronto, ON, Canada; ^6^ Joint Department of Medical Imaging, Mount Sinai Hospital, Toronto, ON, Canada; ^7^ Thoracic Surgery, Unity Health Network, St Joseph’s Hospital Site, Toronto, ON, Canada

**Keywords:** Epstein Barr virus (EBV), inflammatory follicular dendritic cell sarcoma, mutational profile, STAT3, chemotherapautic

## Abstract

EBV-positive inflammatory follicular dendritic cell sarcoma (EBV+ IFDCS) is an uncommon disease primarily observed in Asia. It is characterized by the development of tumors believed to originate from follicular dendritic cells (FDC). The consistent association between this condition and clonal EBV infection suggests EBV’s involvement as an etiological factor. However, diagnosing EBV+ IFDCS can be challenging due to its morphological variability and diverse immunohistochemical staining patterns. The genetic characteristics of EBV+ IFDCS remain insufficiently understood. To address this knowledge gap, we present a case study of a 47-year-old male patient diagnosed with EBV+ IFDCS. We utilized a Next-generation sequencing (NGS) platform to investigate the genetic profile of the tumor cells. We identified a single pathogenic mutation (G618R) in the *STAT3* gene. This finding provides valuable insights into the genetic alterations associated with EBV+ IFDCS and potentially contributes to our understanding of the disease’s pathogenesis.

## Introduction

EBV-positive inflammatory follicular dendritic cell sarcoma (EBV+ IFDCS) is a rare condition predominantly found in Asia, in which the tumor cells are believed to arise from follicular dendritic cells (FDC) (1). It almost exclusively occurs in liver or spleen although more cases of extrahematosplenic location have been reported (2–4). Morphologically, neoplastic cells resemble normal follicular dendritic cells, with spindly cells and cytoplasmic dendrites, but may also present with atypical features, such as enlarged, frequently folded, and hyperchromatic nuclei (5). Tumor cells typically exhibit immunophenotypic characteristics of FDCs, such as the expression of CD21, CD23, CD35, D2-40, EGFR, and CXCL13. However, they may also exhibit myoid cell differentiation (5). A notable histological distinction from FDCS is the presence of a prominent reactive lymphoplasmacytic infiltrate, consisting of mixed B and T cells (1, 6, 7). The condition is consistently associated with clonal EBV infection (7). The disease primarily affects the liver or spleen, although it may rarely involve other organs. EBV+ IFDCS is generally indolent, but intra-abdominal recurrences may occur. Its distinct clinicopathological features of paucity of tumor cells, strong reactive lymphoid infiltration and EBV positivy set it apart from the more common and typically more aggressive FDCS, thus warranting a separate diagnosis (8). The genetic features of EBV+ IFDCS are not well-established.

Here, we present a unique case of EBV+ FDCS in which we identified a hotspot mutation in the *STAT3* gene, which has not been previously reported in these tumors. Understanding the role of this mutation in EBV+ IFDCS pathogenesis, while not specifically required for diagnosing, could potentially pave the way for the development of targeted therapies tailored specifically to patients with this genetic alteration.

## Case presentation

**Figure 1:**
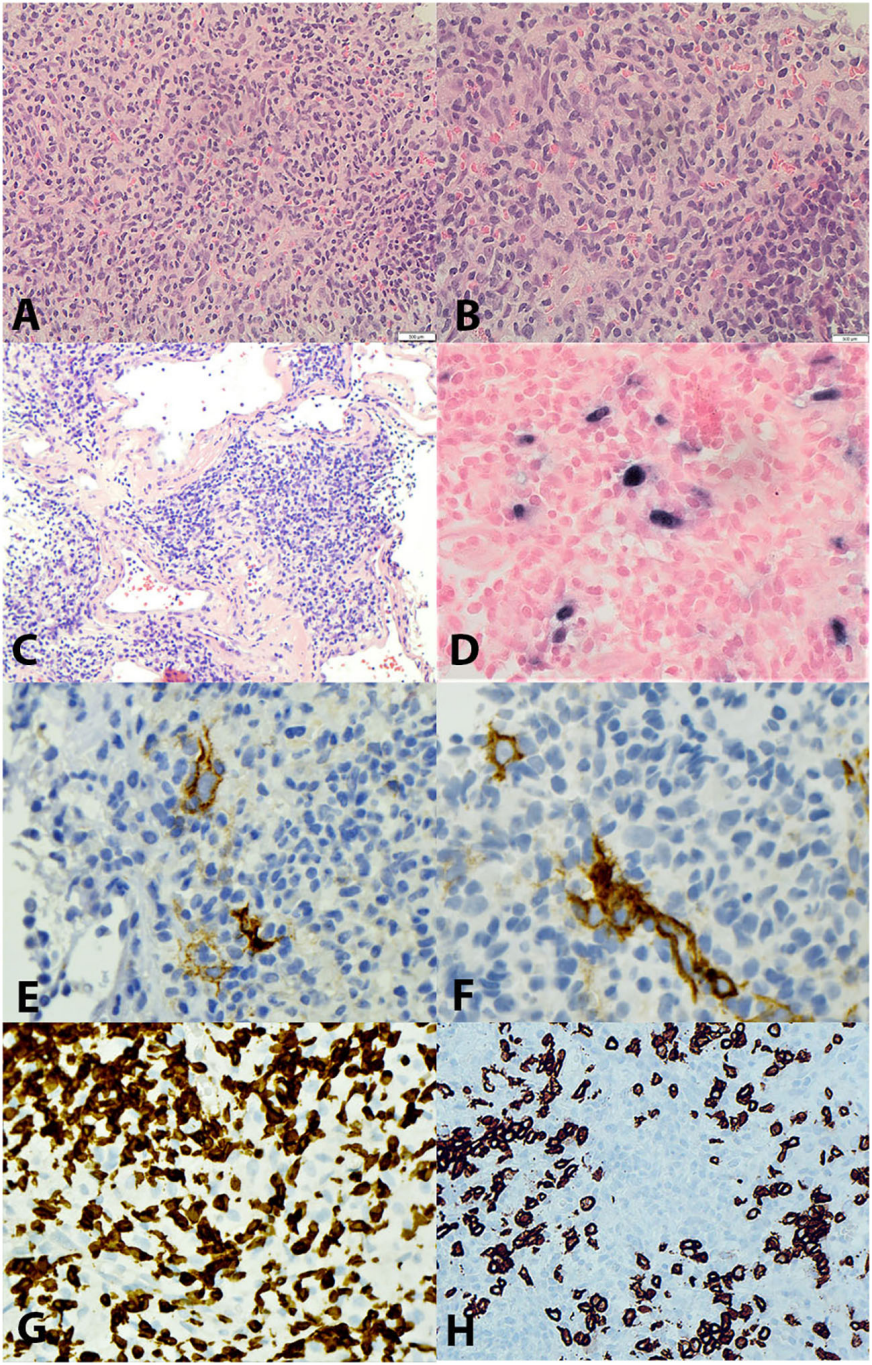
**(A)** Hematoxylin & Eosin (H&E), 20X and **(B)** 40X showing scattered spindled tumour cells with round to oval nuclear membrane, slightly decondensed chromatin and with prominent nucleoli in the stroma with prominent mixed inflammatory cells of predominantly small lymphocytes. **(C)** H&E, 40X highlighting the large vessels with pink hyaline degeneration. **(D)** EBER-by *in-situ* hybridization, 40X, positive in tumour cells. (**E**, **F**) CD21, 40X, positive in occasional tumour cells. **(G)** CD3, 40X, positive in many small T lymphocytes. **(H)** CD20, 40X, positive in small B lymphocytes, tumour cells are negative.

A previously healthy 47-year-old Asian man presented with dysphagia, left-sided epigastric discomfort, and worsening back pain. Clinical examination revealed a palpable fullness in the epigastric area. Computed tomography (CT)- guided imaging demonstrated esophageal thickening, upper abdominal and retroperitoneal lymphadenopathy, and a soft tissue mass measuring 10.4x10.0 cm in the left upper quadrant of the abdomen abutted the medial border of the left lobe of the liver and the lesser curvature of the stomach. An endoscopy revealed a 1.6 cm lesion in the esophagus. The biopsy showed a squamous cell carcinoma with positivity for CK5, p63, and p40 while *in situ* hybridization for Epstein-Barr virus-encoded small RNA (EBER) was negative. The biopsy of the left upper quadrant mass, however, revealed an apparently unrelated neoplasm with a heterogeneous infiltrate consisting mostly of small to intermediate-sized lymphocytes, histiocytes, and rare plasma cells interspersed with scattered neoplastic large spindle to ovoid cells with prominent nucleoli which only comprised approximately 5% of the cellular constituents (**Figures 1A–C**). These large neoplastic cells exhibited focal positivity for CD21 (**Figures 1E, F**) and strong expression of Epstein-Barr virus-encoded small RNA (EBER) by *in situ* hybridization (**Figure 1D**). The proliferation index was low in the overall cell population but was interpreted to be approximately 90% in the neoplastic cells. These cells did not show expression of other markers associated with follicular dendritic or fibroblastic cell differentiation (CD23, D2-40, SMA) or markers for lymphoid (CD1a, CD3 (**Figure 1G**), CD4, CD8, CD10, CD15, CD19, CD20 (**Figure 1H**), CD30, CD79a, CD138, BCL6, OC2, PD-1, MUM1, PAX5), myeloid (CD33, CD34, CD56), histiocytic, (CD68, CD163, ALK-1, S100), and epithelial cells (PAN-CK). Ziehl-Neelsen (ZN) and Grocott’s Methenamine Silver (GMS) staining were negative for microorganisms. Immunohistochemistry for human herpesvirus 8 (HHV-8) yielded negative results. The majority of the lymphoid cells observed were CD3-positive T cells, with a slight predominance of cytotoxic CD8+ T cells. Given the co-occurrence of EBV+ IFDCS and esophageal squamous cell carcinoma and the uncertainty surrounding lymph node involvement, a multidisciplinary decision was made to initiate systemic therapy with Gemcitabine and docetaxel to address both conditions. Subsequently, definitive therapy, comprising surgery and/or radiotherapy for both lesions, was planned. The patient successfully completed six cycles of gemcitabine and docetaxel therapy. A restaging CT scan demonstrated a reduction in the size of both the liver mass and the esophageal lesion, while lymphadenopathy remained stable. Representative images of the lesion on CT at diagnosis and post-therapy are shown in **Supplementary Figure 1**. The timeline of clinical events is outlined in **Supplementary Figure 2**.

## Molecular analysis

**Figure 2:**
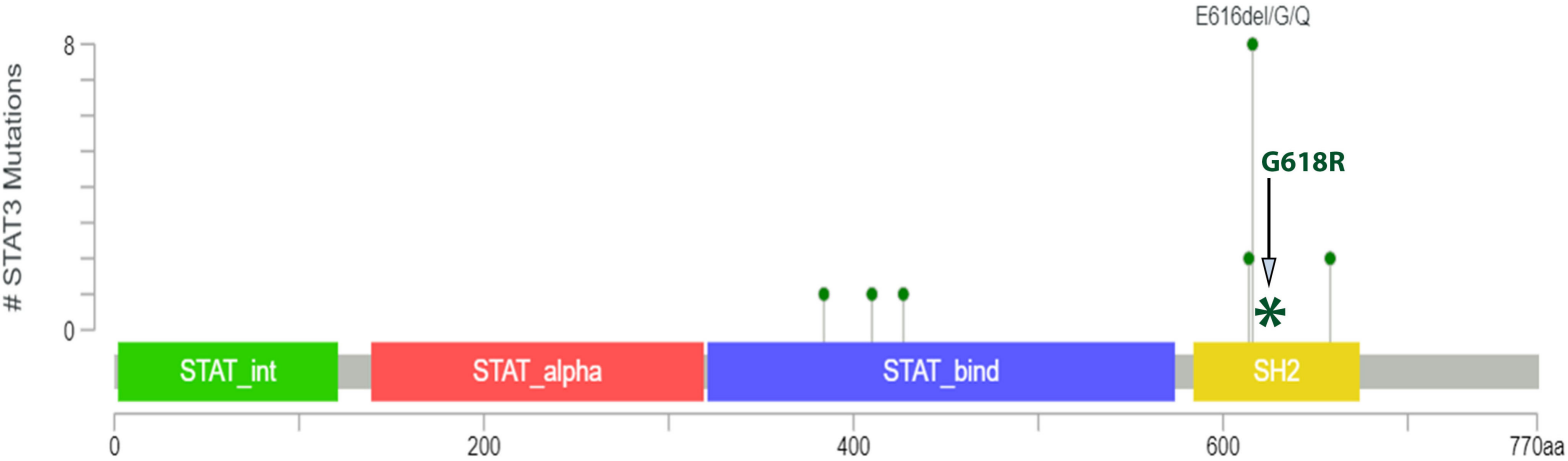
The lollipop plot shows oncogenic mutations of the *STAT* gene by Zehir et al. (11) in which they performed Targeted sequencing of 10,000 clinical cases using the MSK-IMPACT assay. This shows 10 mutational variants present within the SH2 domain. The coiled-coil domain is represented by STAT_alpha and the DNA binding domain is represented by STAT_bind. The image is taken from the OncoKB™ database. The site of the *STAT3* p.Glu618Arg missense mutation identified in this case is represented by an Asterick in the above image.

Clonality analyses using the BIOMED-2 Assay (9) were inconclusive/indeterminate due presumably to degraded nucleic acid or the presence of an inhibitor. NGS analysis using the Illumina TruSight™ Oncology 500 (TSO500) (10) (**Supplementary Table 1**) revealed a relatively stable genome with 1.6 mutations per megabase and no evidence of microsatellite instability. Besides the detection of 17 variants of potentially germline origin (**Supplementary Table 2**) a single variant of STAT3 (NM_139276.3): c.1852G>C (p.Glu618Arg) was detected with a VAF of 1.6%, which is presumed to be from the neoplastic population (<5% tumour cells). In addition, the variant was observed in 10 reads (of a total of 621 reads) and was not detected in a validation set of 72 solid tumour samples reducing the likelihood of an analytical artifact in the sequencing. This variant is a missense change in the SH2 domain of the STAT3 gene. The SH2 domain is a site of other oncogenic mutations as shown in **Figure 2**. Functional studies have shown this mutation to be associated with increased phosphorylation of protein and enhanced growth activity thus representing a gain of function (12, 13). It has been observed previously in lymphoid cancers. Importantly this variant has not been identified in the normal population database (GnomADv2.11). Based on the functional evidence and recurrence in other cancers, we consider this a driver of follicular dendritic cell sarcoma; however, the clinical significance remains unclear.

## Discussion

Genetic alterations in FDCS and EBV+ IFDCS have not been extensively investigated, but more research has been conducted on the molecular changes associated with FDCS. Among the frequently observed mutations in FDCS, several affect the NF-KB pathway, including genes such as *BIRC3*, *NFKBIA*, *TRAF3*, *TNFAIP3*, *SOCS3*, and *CCND2*. In addition, mutations in *CDKN2A* and *TP53* have been reported in FDCS (14, 15). In rare cases of FDCS, *BRAF* V600E mutations have been reported (16). Additionally, copy number variations involving the *JAK2* gene have been observed in some instances (17). Limited information is available regarding the mutational changes in EBV+ IFDCS due to the rarity of these tumors. In one study, *BRAF* V600E mutation was detected in 2 out of 5 cases (16). Furthermore, only three cases of EBV+ IFDCS have undergone NGS analysis as reported in the literature. Among these cases, one had two variants identified in the *BCORL1* and *JAK2* genes, which were predicted to be germline and non-pathogenic (14). In another study involving two cases, NGS analysis revealed a variant of the *RICTOR* gene in both cases (7). The 500-gene panel used in this study encompassed all the genes mentioned earlier, yet no pathogenic variants of these genes were detected in this particular tumour.

A thorough analysis of the COSMIC database showed that the same *STAT3* mutation identified in our case has also been detected in 21 other samples. The STAT3 mutation has been found in somatic databases (COSMIC, TCAG) and showed an enrichment in hematological and lymphoid malignancies particularly T-cell lymphomas (18). The variant also contains two submissions in ClinVar including one with peripheral T-cell lymphoma (PMID: 22859607). Notably, none of the cancer types described in these databases included EBV-Positive Inflammatory Follicular Dendritic Cell Sarcoma. Although our study does not conclusively evaluate the functional effect of the *STAT3* variant, *in silico* programs such as AlignGVGD, Mutation Taster, PolyPhen and SIFT unanimously predict the variant to be deleterious.

Moreover, *STAT3* mutations have also been implicated in other types of sarcoma, such as Ewing family sarcomas (19) and Kaposi Sarcoma (20). *STAT3* (Signal transducer and activator of transcription 3) gene belongs to the family of *STAT* genes. These genes serve as transcription factors that regulate the activation or inhibition of target genes by binding to their regulatory regions. *STAT3* specifically plays a significant role in cell proliferation, migration, and apoptosis, and it interacts with Janus kinases (JAKs), epidermal growth factor receptor (EGFR), and interleukin 6 (IL6) (21).

Previous studies have demonstrated that *STAT3* alterations are involved in oncogenesis and cancer progression with constitutive activation of the JAK/STAT pathway observed in many solid tumours (21, 22).

## Conclusion

This case underscores the challenges in diagnosing EBV+ IFDCS primarily due to the scarcity of tumour cells masked by the presence of a brisk inflammatory infiltrate. To identify the atypical cells expressing markers of follicular dendritic cells, an extensive immunohistochemical panel is often necessary, with the definitive confirmation often relying on positive results for EBER-ISH. Moreover, this case contributes valuable information to the existing literature by presenting a complex scenario involving a second unrelated malignancy and lymphadenopathy, which could potentially be attributed to either tumours. While the majority of EBV+ IFDCS cases are typically managed with surgery alone, the inclusion of adjuvant chemotherapy, specifically gemcitabine and docetaxel, has shown promising responses. Traditional CHOP chemotherapy regimens have demonstrated limited success in FDCS treatment, likely due to FDCS originating from mesenchymal rather than hematopoietic origins. On the other hand, FDCS cases treated with sarcoma-directed regimens like gemcitabine and docetaxel have exhibited improved outcomes compared to CHOP (23). Notably, there is a reported case of metastatic EBV+ IFDCS that achieved a complete clinical response, as indicated by imaging studies, following treatment with gemcitabine, docetaxel, and radiotherapy (24). Although the NF-KB pathway appears to be the primary molecular pathway affected in FDCS cases, alterations potentially impacting the JAK/STAT pathway have also been observed, including two cases with *JAK2* copy number variations and our case showing *STAT3* mutation. While it may not be deemed essential for the diagnosis of this condition, given the unique morphological and phenotypic characteristics, understanding the underlying disease biology remains valuable. As recently reviewed, STAT3 protein has a significant role in life cycle and pathogenesis of EBV (25). STAT3 activation occurs rapidly after infection and contributes to various cancer-related properties of EBV, including cell proliferation, invasion, and angiogenesis. Additionally, STAT3 plays a critical role in blocking the DNA damage response, which aids in the establishment of viral latency and influences the transition between latent and lytic phases. This dual function of STAT3 ensures the persistence of EBV in the host. Understanding the complex interplay between STAT3 and these viruses provides valuable insights that may have therapeutic implications for combating EBV-related diseases and cancer (**Supplementary Figure 3**). The discovery of *STAT3* in this tumor type represents a novel finding, albeit with limited reported cases of EBV+ IFDCS in the literature with molecular analysis which makes it challenging to identify common mutation patterns within this unique subgroup of tumors, as well as in comparison to FDCS. While the *STAT3* mutation in our case, which we believe to be genuinely representative of the neoplastic population in our sample, appears to be prevalent in hematopoietic proliferation, it is important to recognize that EBV+ IFDCS remains a mesenchymal neoplasm with a better response to sarcoma-directed systemic therapies rather than hematopoietic-directed therapies.

To fully understand the significance of the *STAT3* G618R hotspot mutation in our case and to explore the genetic landscape of this disease entity, it would be imperative to conduct molecular testing on a large cohort of EBV+ IFDCS cases.

## Data availability statement

The raw data supporting the conclusions of this article will be made available by the authors, without undue reservation.

## Ethics statement

The studies involving humans were approved by University Health Network Review Ethics Board. The studies were conducted in accordance with the local legislation and institutional requirements. The participants provided their written informed consent to participate in this study. Written informed consent was obtained from the individual(s) for the publication of any potentially identifiable images or data included in this article.

## Author contributions

MR: Conceptualization, Data curation, Investigation, Validation, Writing – original draft, Writing – review & editing. PS: Data curation, Investigation, Writing – review & editing, Formal Analysis, Methodology. GW: Data curation, Investigation, Writing – review & editing. TC: Data curation, Investigation, Writing – review & editing. MK: Data curation, Investigation, Writing – review & editing. AS: Data curation, Investigation, Writing – review & editing, Conceptualization, Funding acquisition, Supervision, Validation, Writing – original draft.

## Supplementary material

Supplementary Figure 1 Serial contrast-enhanced CTs of the upper abdomen in the portal venous phase show a large solid and heterogeneous mass (arrows) centred in the gastrohepatic ligament. The lesion is separate from the liver and the adjacent stomach and measured greater than 10.4 x 10.0 cm in the baseline study. An interval decrease in the size of the lesion was appreciated at 3 and 5 months post-commencement of Gemcitabine and docetaxel.

Supplementary Figure 2 A timeline of events from the presentation of symptoms in October 2022, diagnostic pathway, the commencement of treatment and current status post 6 cycles of systemic therapy.

Supplementary Figure 3 Complex interaction between EBV and STAT3 in lympohcytes. EBV binding/entry rapidly activates STAT3 via JAK protein. STAT3 can also be activated at Y705 by LMP1 in cooperation of cellular proteins. STAT3 can transcriptionally activate LMP1 and cellular PCBP2; PCBP2 represses EBV lytic genes. EBV: Ebstein-Barr virus; LMP1: latent membrane protein 1; PCBP2: poly(rC)-binding protein 2; STAT3: signal transducer and activator of transcription 3 ((adapted from reference # (25)).
